# Adrenergic nerves regulate B cell responses to large antigens through modulation of lymph node permeability

**DOI:** 10.21203/rs.3.rs-9931614/v1

**Published:** 2026-06-29

**Authors:** Maureen Cox, Jessica Reel, Jumana Abbadi, Sydney Honold, Happy Agarwal, Rohit Singh, Jacob Farriester, Hunter Abrahamsen, Tor Olofsson, Melissa Testut, Kaitlyn Pipkin, Jordan May, Katarzyna Cizio, A. Bueno, Rodney Tweten, Mark Lang, Lacey McNally

**Affiliations:** University of Oklahoma Health Science Center; University of Oklahoma Health Science Center; University of Oklahoma Health Science Center; University of Oklahoma Health Science Center; University of Oklahoma Health Science Center; University of Oklahoma Health Science Center; University of Oklahoma Health Science Center; University of Oklahoma Health Science Center; University of Oklahoma Health Science Center; University of Oklahoma Health Science Center; University of Oklahoma Health Science Center; University of Oklahoma Health Science Center; University of Oklahoma Health Science Center; University of Oklahoma Health Science Center; University of Oklahoma Health Science Center; University of Oklahoma Health Science Center; University of Oklahoma Health Science Center

## Abstract

Adrenergic nerves innervate all secondary lymphoid organs, although the role of these nerves in adaptive immune responses is unclear. Systemic depletion of these nerves compromises germinal center formation after infection or vaccination. We have found that these nerves locally are required for optimal B cell responses to the large antigen keyhole limpet hemocyanin (KLH), but are dispensable for the B cell response to the small antigen Ovalbumin. After vaccination or infection the reticulin fiber network in the lymph node undergoes remodeling to allow high molecular weight particles, such as KLH, access to the conduit system when they are ordinarily restricted. In contrast, low molecular weight molecules like Ovalbumin always have access to the conduit system. This remodeling of the reticulin fiber network to allow permeability to large molecules was dependent upon the local adrenergic nerves. These findings may help explain why patients with nerve injuries are more likely to have recurrent infections.

## Teaser

Stress nerves remodel the lymph node after vaccination or infection to allow immune responses.

## Introduction

The conduit system in the lymph node (LN) is a reticulin fiber network that allows rapid dissemination of antigens and molecules smaller than 70kDa^1, 2^. The conduit system itself is capable of moving much larger macromolecules^3^ and undergoes extensive remodeling during immune responses^4^, however whether this system gains permeability to large draining antigens is unknown. Here, we describe a role for the adrenergic nerves^5^ in remodeling reticulin fiber structures in the LN and rendering the conduit system permeable to large antigens draining from a site of vaccination. Nerve depleted LNs remain impermeable to large molecules, have poor B cell responses specifically to large antigens, and have poor accumulation of large antigens in germinal centers. These findings are relevant to patients with peripheral nerve injuries or dysfunction, as these patients are highly susceptible to recurrent infections suggesting defects in immune memory^6, 7, 8^.

## Results

**Figure 1:**
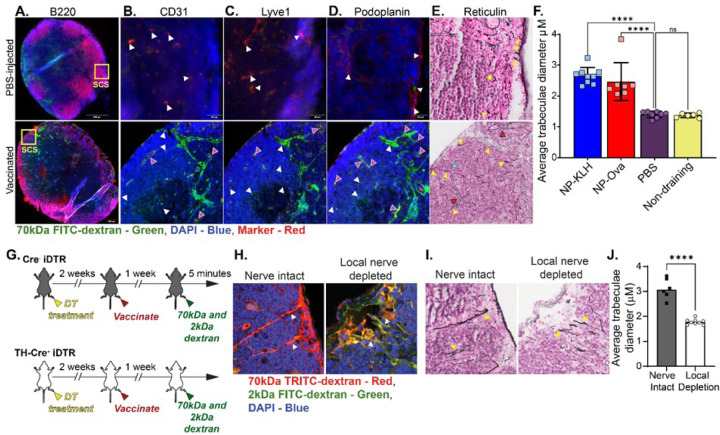
Vaccination renders the conduit system permeable to high molecular weight dextrans in an adrenergicnerve dependent manner. C57Bl6 mice were injected with either PBS, NP-Ova in alum, or NP-KLH in alum in the footpad. On day 7 post-vaccination or PBS-injection, mice were injected with 70kDa FITC-dextran in the footpad 5 minutes prior to euthanasia. **A.**Distribution of HMW dextran (green) and B220 (red) in the lymph node 7 days after PBS or NP-Ova vaccination, counterstained with DAPI (blue). Boxes mark areas of interest near the subcapsular sinus and the B cell follicles (SCS). **B-D.**The SCS region of interest identified in **A**, stained for anti-CD31 (**B**, red), Anti-LYVE-1 (**C**, red), or anti-podoplanin (**D**, red). Green staining in all sections is 70kDa FITC-dextran. All sections are imaged at 20x magnification. White arrows indicate areas of positive antibody staining. Pink arrows indicate areas of co-localization with FITC-dextran. **E.** Serial section to **D**, stained for reticulin fiber (black) and counterstained with nucleofast red. Yellow arrows indicate putative trabeculae and conduits which were quantified for diameter. Red arrows indicate putative blood vessels that were excluded from quantification. Blue arrows indicate conduits that were also excluded from quantification. **F.** Average trabeculae diameter on day 7 postvaccination in PBS-draining and vaccine-draining lymph nodes. Symbols represent average trabeculae diameter in lymph nodes from individual mice. Composite of 3 experimental cohorts, symbols represent individual mice. All analyses used C57Bl6 mice purchased from Jackson Laboratories (000664). *p<0.05, **p<0.01, ***p<0.001, ****p<0.0001. **G.** Schematic for local nerve-depletion, vaccination, and dextran administration in TH-cre iDTR mice and Cre- controls. DT treatment, vaccination, and dextran were all administered in the same footpad at the indicated times. **H.** 70kDa TRITC-dextran (red), 2kDa FITCdextran (green), and DAPI (blue) staining in draining popliteal lymph nodes 7 days post-vaccination. White arrows indicate regions of any size dextran penetration into the lymph node from the subcapsular sinus. **I.** Serial section to **H**, stained for reticulin fiber (black) and counterstained with nucleofast red (red). Yellow arrows indicate reticulin fiber structures that correspond to regions of dextran penetration identified in **H**. J. Quantification of putative trabeculae diameter in nerve-intact and locally nerve depleted lymph nodes 7 days post-vaccination. Representative or composite of two experimental cohorts, symbols represent individual mice, ****p<0.0001.

We vaccinated C57Bl6 mice with 4-Hydroxy-3-nitrophenylacetyl (NP) conjugated to a protein carrier in alum in the footpad or injected with PBS alone. To determine the size limit of molecules entering the lymph node 7 days post-vaccination, mice were injected with > 70kDa FITC-labeled dextran in the footpad 5 minutes prior to euthanasia and fixation of the popliteal LN. In LNs draining a PBS injection, FITC-dextran is restricted to the subcapsular sinus and medullary sinus (Fig. 1A–D, Extended Data Fig. 1A-D). Vaccination renders the LN permeable to the large dextran, with FITC-dextran staining penetrating into the LN from the subcapsular sinus (SCS) and interspersed in the B cell follicles (Fig. 1AD, Extended Data Fig. 2A-E). FITC-dextran was present in fine hair-like staining, characteristic of the conduit system, as well as larger channels that often appeared connected to the SCS interspersed with B220 + B cell follicles (Fig. 1B–D, Extended Data Fig. 2A-E). We counterstained the dextran-labeled tissue sections with either CD31, LYVE-1, or Podoplanin to identify blood endothelium, lymphatic endothelium, and fibroblastic reticular cells (FRC), respectively. The FITC-dextran staining in the LN was not surrounded by CD31 + staining, indicating this dextran is not localized inside of blood vessels (Fig. 1B, Extended Data Fig. 1A and 2C). While we do observe FITC-dextran staining associated with LYVE-1 staining in the medullary sinus (Extended Data Fig. 1C), the FITC-dextran channels penetrating from the SCS in vaccinated LNs are not associated with LYVE-1 (Fig. 1C, Extended Data Fig. 2D). Instead these channels are frequently near podoplanin+ FRCs (Fig. 1D, Extended Data Fig. 2E). The FRC network is responsible for producing the reticulin fiber conduit system within the LN^4^.

We stained serial sections of dextran-treated LNs for reticulin fiber and evaluated for correspondence to the areas of FITC-dextran accumulation. Both large reticulin fiber structures and narrow reticulin fiber conduits co-localize with FITC-dextran in serial sections (Fig. 1D,E). The large reticulin fiber structures were connected to the subcapsular sinus adjacent to the B220 + B cell follicles and corresponded to the same areas we observe FITC-dextran penentrating from the SCS (Fig. 1D, Extended Data Fig. 2F). The diameter of these large reticulin fiber channels was quantified after vaccination with either NPOvalbumin (NP-Ova) or NP-Keyhole Limpet Hemocyanin (NP-KLH), or after PBS injection and in nondraining popliteal LNs. Only reticulin fiber structures larger than 0.5μM in diameter were included in this analysis. Structures that appeared to be blood vessels or lymphatic vessels based on the pattern and location of reticulin fiber staining were also excluded (Extended Data Fig. 2F). These structures postvaccination had a loose net-like appearance (Fig. 1E, Extended Data Fig. 2F), and an average diameter of 2.4 to 2.6 μM (Fig. 1F). In PBS-draining LNs these structures had a compact dark appearance (Fig. 1E, upper), with an average diameter of 1.39μM, similar to the diameter in non-draining LNs (Fig. 1F). Both the location in the LN and the increased diameter compared to conduits are consistent with these structures being trabeculae^9, 10^. Herpes virus infection also drives trabeculae remodeling in a similar manner^11^. Thus remodeling of these trabeculae is likely a conserved attribute of responding LNs.

Trabeculae are innervated^12, 13^; we tested whether adrenergic nerves are required for trabeculae remodeling and conduit permeability to HMW dextran after immune challenge. C57Bl6 mice were either mock-treated or chemically depleted of adrenergic nerves with 6-hydroxydopamine (6OHDA)^14^ 2 weeks prior to either vaccination or infection with *Staphylococcus aureus* in the footpad. Seven days postchallenge mice were injected with a mix of 2kDa-FITC and 70kDa-TRITC dextran in the footpad (Extended Data Fig. 3A). In nerve-intact cohorts, both 2-kDa FITC-dextran (green) and 70kDa TRITC-dextran (red) penetrated from the SCS into the LN cortex 7 days post-vaccination or infection (Extended Data Fig. 3B,C). In contrast, in nerve depleted cohorts the 70kDa TRITC-dextran was localized in the subcapsular sinus, and excluded from the cortex after either vaccination or infection (Extended Data Fig. 3B,C). In these nerve-depleted cohorts, the 2kDa dextran still readily accesses the conduit system of the LN (Extended Data Fig. 3B,C). Serial sections were stained for reticulin fiber. In the nerve intact LNs, these reticulin fiber structures had a wide net-like appearance and colocalized to the areas we observe the 70kDa TRITC-dextran enter the node (Extended Data Fig. 3B,C). The average reticulin fiber diameter after vaccination or infection in nerve intact mice was 2.26μM and 2.35μM, respectively (Extended Data Fig. 3D,E). In nerve depleted cohorts, reticulin fiber had a compact “closed” conformation, with an average trabeculae diameter of 1.53–1.56 μM (Extended Data Fig. 3D,E), irrespective of vaccination or bacterial challenge.

The adrenergic nervous system drives numerous physiological responses, including systemic changes to blood pressure and heart rate, and release of glucocorticoids from the adrenal glands^15^. The total removal of the adrenergic nerves with 6OHDA could impact the LN indirectly due to systemic alterations. We adopted a local depletion method^16^ to eliminate adrenergic nerve fibers locally in the draining LN. All adrenergic nerve fibers express the enzyme tyrosine hydroxylase (TH). We generated animals where the diphtheria toxin (DT) receptor is expressed in TH^+^ cells (TH-Cre iDTR). These animals were treated with 0.1 ng/g of DT in one footpad for two treatments, 2 days apart. Control Cre^−^ mice treated with DT (Fig. 1G). This dose of DT resulted in deletion of adrenergic nerve fibers in the draining popliteal LN, but not in the draining inguinal LN or the non-draining popliteal LN (Extended Data Fig. 4A,B). Approximately 2–4% of macrophages express TH (Extended Data Fig. 4C)^17, 18^; although DT treatment eliminates these TH+ immune cells in the LN 48 hours post-treatment, the percentage of TH+ macrophages in the LN recover by 2 weeks post-depletion (Extended Data Fig. 4C).

Both TH-iDTR and cre^−^ iDTR animals were treated with DT 2 weeks prior to vaccination with NP-Ova in alum in the treated footpad (Fig. 1G), and the vaccinated footpad injected with a mix of 70kDa TRITC dextran and 2kDa FITC dextran 7 days post-vaccination. In neurologically intact mice, 70kDa TRITCdextran accumulated in large channels from the subcapsular sinus and in conduits in the B cell follicles (Fig. 1H, Extended Data Fig. 5A,B). In serial sections these large areas of TRITC-dextran staining often appeared to contain the open, net-like reticulin fiber structures of the putative trabeculae (Fig. 1H, Extended Data Fig. 5C,D). After vaccination in these nerve-intact mice the average putative trabeculae diameter was 3.11μM (Fig. 1I). In contrast, with the local nerve depletion, 70kDa TRITC-dextran was localized to the subcapsular sinus, while the 2kDa FITC-dextran was able to access the conduit system and the thicker fibers that are consistent with closed trabeculae (Fig. 1H, Extended Data Fig. 5A,B). In serial sections, the putative trabeculae in the nerve-depleted LN appear to have the compact “closed” conformation (Fig. 1H, Extended Data Fig. 5C,D), and have an average diameter of 1.78μM (Fig. 1I). The physical expansion of the LN is thought to play a role in the remodeling of the conduit system during immune responses^4^, however we did not observe differences in the mass or cellularity of nerve-intact and nerve-depleted LNs post-vaccination (Extended Data Fig. 4D,E).

**Figure 2:**
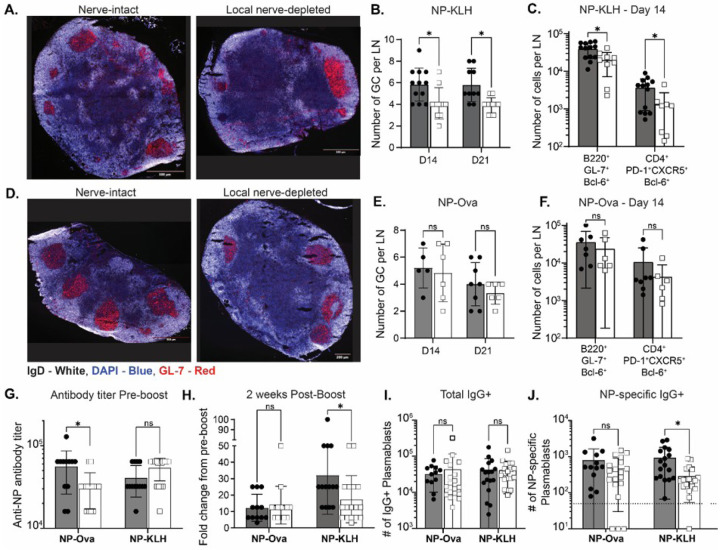
Adrenergic nerves in the lymph node are required for optimal B cell responses to NP-KLH, not NP-Ova. Neurologically intact and locally depleted TH-iDTR mice were vaccinated in the treated footpad with either NP-KLH or NP-Ova absorbed in alum. **A.** Representative sections from NP-KLH draining popliteal lymph nodes 14 days post-vaccination stained for DAPI (blue), IgD (white) and GL7 (red). **B.** Quantification of the number of germinal centers identified across 4–6 discontinuous sections. Symbols represent individual mice, composite of 3 experimental cohorts. **C.** The number of B220+ GL7+ Bcl6+ germinal center B cells and CD4+ CXCR5+ Bcl6+ T follicular helper cells quantified by flow cytometry 14 days post-NP-KLH vaccination. **D**. Representative sections from NP-Ova draining popliteal lymph nodes 14 days post-vaccination stained for DAPI (blue), IgD (white), and GL7 (red). **E.** Quantification of the number of germinal centers identified across 4–6 discontinuous sections. Symbols represent individual mice, composite of 2 experimental cohorts. **F.** The number of B220+ GL7+ Bcl6+ germinal center B cells and CD4+ CXCR5+ Bcl6+ T follicular helper cells quantified by flow cytometry 14 days post-NP-Ova vaccination. **G.** The anti-NP antibody titer in the serum determined by ELISA 28 days post-vaccination with either NP-Ova or NP-KLH. **H.** On day 28 post-vaccination, after blood collection mice from (**G**) were boosted in the contralateral neurologically intact footpad. Mice were boosted with the same antigen as priming. The anti-NP antibody titer was determined 2 weeks post-boost, and the fold change from the titer on day 28 determined. **I.** The total number of IgG+ plasmablasts in the bone marrow was determined in all mice 5 weeks post-boost by Elispot. **J.** the total number of IgG+ NP-specific plasmablasts was determined 5 weeks post boost by Elispot. Symbols represent individual animals. **G-J** are a composite of 3 experimental cohorts. All analyses used TH-iDTR mice as well as cre- littermates treated with DT, Cre+ iDTR- littermates treated with DT, or TH-iDTR mice treated with PBS. *p<0.05.

The conduit system can concentrate small antigens in B cell follicles, however it is not well understood how large antigens gain access to the LN during a primary infection. We hypothesized that this enhanced permeability of the LN to large molecules would be important for B cell responses to large antigens, while small antigens would still access the conduit system. The germinal center response to both *S. aureus* (~ 100 gigadaltons) and KLH (450kDa) is impaired in chemically sympathectomized mice^14, 19^. In contrast, Ova is only 45kDa, and can therefore access the conduit system without any remodeling. Nerve-intact and locally nerve-depleted TH-iDTR mice were vaccinated with either NP-Ova or NP-KLH. Local depletion of adrenergic nerves compromised the formation of germinal centers in the draining popliteal LN following vaccination with NP-KLH (Fig. 2A,B), corroborating published studies utilizing systemic depletion^19^. The number of GL-7^+^ Bcl6^+^ germinal center B cells and CXCR5^+^ PD-1^+^ Bcl6^+^ CD4^+^ T follicular helper cells were also reduced in the draining LN 14 days post-vaccination with NP-KLH (Fig. 2C, Extended Data Fig. 6A,B). In contrast, local nerve depletion did not impair the germinal center response to vaccination with NP-Ova either 14 or 21 days post-vaccination (Fig. 2D,E). The total number of GL-7^+^ Bcl6^+^ germinal center B cells and CXCR5^+^ Bcl-6^+^ CD4^+^ T follicular helper cells in the draining popliteal LN after vaccination with NP-Ova was not impacted by the loss of innervation (Fig. 2F).

Germinal center responses are critical for the generation of memory B cells following vaccination or infection. Memory B cells preferentially form antibody secreting cells after boosting^20, 21, 22, 23, 24, 25^, promoting robust secondary antibody titers. We measured the anti-NP IgG antibody titer in nerve-intact and nerve-depleted mice after vaccination with either NP-Ova or NP-KLH. There was no defect in the antiNP IgG antibody titer in nerve-depleted cohorts 28 days after vaccination with NP-KLH (Fig. 2G), supporting our previous findings following Staphylococcus aureus infection^14^. A minor defect in the antiNP antibody titer was observed in nerve-depleted NP-Ova vaccinated mice 28 days post-vaccination (Fig. 2G). Mice were boosted with the same antigen as priming in the contralateral neurologically-intact footpad on day 28 post-vaccination, and the increase in antibody titer from day 28 determined by ELISA 14 days post-boost. Nerve depletion did not impact the ability to boost NP-Ova vaccinated animals, however the recall antibody response in NP-KLH vaccinated cohorts was blunted if cells were primed in the absence of adrenergic nerves (Fig. 2H). Neuronally intact controls responded similarly, irrespective of TH-cre expression (Extended Data Fig. 6C,D). These results indicate the nerve-dependent change in lymph node permeability to large antigens significantly impacts the humoral response.

We found no change in the total number of IgG-producing plasmablasts in the bone marrow of nervedepleted mice 5 weeks post-boost, indicating that local nerve depletion did not broadly impact the generation or retention of plasma cells in the bone marrow (Fig. 2I). Further, nerve-depletion did not impact the number of NP-specific IgG-producing plasma cells after NP-Ova vaccination (Fig. 2J). However, we observed a significant reduction in the number of NP-specific plasma cells in nervedepleted cohorts vaccinated with NP-KLH (Fig. 2J). Collectively, these results demonstrate that local adrenergic nerves are critical for B cell responses to the large antigen NP-KLH, but are dispensable for the response to the small antigen NP-Ova.

**Figure 3:**
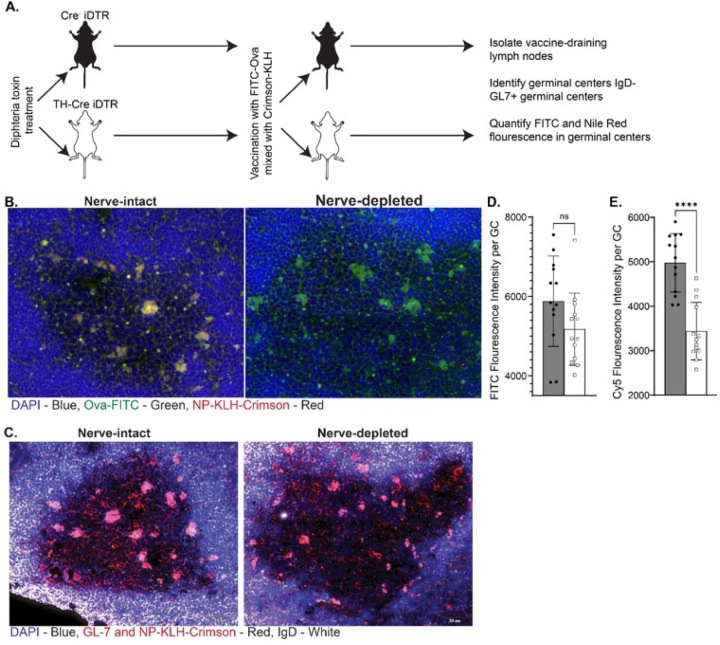
Depletion of local adrenergic nerves impairs accumulation of large antigens in developing germinal centers. **A.** Experimental schematic. Neurologically intact or locally depleted TH-iDTR mice were vaccinated with FITC-Ova and NP-KLH conjugated to Crimson labeled polystyrene beads. Germinal centers were identified in the B cell follicle as areas of lower DAPI staining 7 days post-vaccination, and as IgD- and GL-7+ in serial sections. **B.** Germinal center from a neurologically intact (left) and nervedepleted (right) animal imaged for DAPI (blue), FITC-Ova (green), and Red-KLH (red). **C.** Serial sections of images in **B**, stained for DAPI (blue), IgD (white), and GL7 (red). GL7 stain fluoresces in the same channel as NP-KLH-Crimson. **D.** Fluorescence intensity of FITC staining in putative germinal centers from nerveintact (black circles) and nerve-depleted (white squares) lymph nodes. **E.** Fluorescence intensity of RedKLH staining in putative germinal centers from nerve-intact (black circles) and nerve-depleted (white squares) lymph nodes. Representative of 2 experimental cohorts, n=7 mice. Symbols represent individual germinal centers quantified from one experimental cohort. ****p<0.0001.

Previous studies of the conduit system have identified unique honeycomb-like structures in the B cell follicles that are connected to the follicular dendritic cells (FDCs)^26^. Low molecular weight antigens transit through the conduit system and lead to rapid activation of B cells^2^. This suggests that antigens that can access the conduit system preferentially accumulate in the B cell follicles to promote and sustain the germinal center response. If nerve-dependent reticulin fiber remodeling allows HMW antigens access to the conduit system and deposition in the B cell follicles, then we should observe differences in the accumulation of HMW antigens in developing germinal centers. Nerve-intact and locally nerve-depleted mice were vaccinated with FITC-Ova and KLH conjugated with Crimson microbeads (Red-KLH), and the distribution of FITC-Ova and Red-KLH was evaluated in the LNs 7 days post-vaccination (Fig. 3A). In nerve-intact LNs, both FITC-Ova and Red-KLH accumulate in the B cell follicles in areas with blasting cells, as determined by DAPI staining (Fig. 3B, Extended Data Fig. 7). These areas of blasting cells are GL7 + IgD- germinal centers when stained in serial sections (Fig. 3C, Extended Data Fig. 7). These germinal centers had large clusters of FITC-Ova and Red-KLH compared to non-germinal center areas of the follicle or the T cell areas of the LN (Extended Data Fig. 7). In contrast, the germinal centers of nerve-depleted LNs were dominated by FITC-Ova staining, with a reduction in Red-KLH staining (Fig. 3A, Extended Data Fig. 7). The intensity of FITC-Ova staining in the putative germinal centers was equivalent between nerve-intact and nerve-depleted LNs (Fig. 3D), however the intensity of Red-KLH staining in these germinal centers was substantially reduced in nerve-depleted cohorts (Fig. 3E).

**Figure 4:**
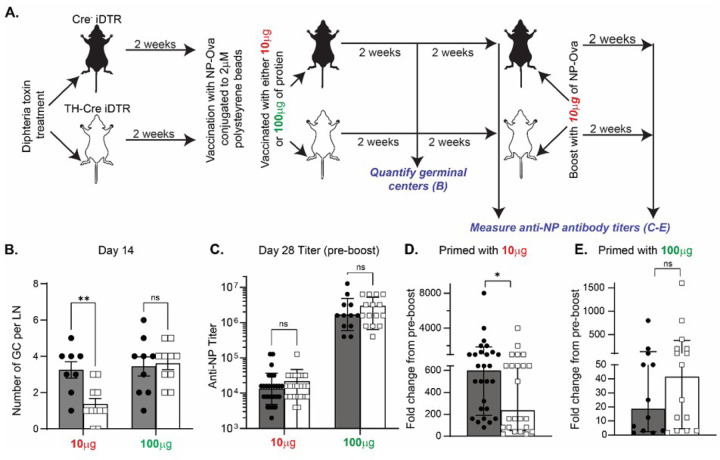
Increasing the dose of antigen in vaccines rescues B cell responses in adrenergic-nerve depleted mice. **A.** Experimental schematic. Nerve-intact and locally nerve-depleted mice were vaccinate with either 10mg or 100mg of NP-Ova conjugated to 2mM polystyrene beads. Germinal center responses quantified14 days post-vaccination. Remaining mice were boosted with 10mg of bead-free NP-Ova in the contralateral footpad 4 weeks post-vaccination, and the anti-NP antibody titer quantified pre- and 2 weeks post-boost. **B.** Quantification of the number of IgD- GL-7+ germinal centers identified across 4–8 discontinuous sections. Nerve intact are black circles, nerve depleted are white squares. Symbols represent individual mice, composite of 2 experimental cohorts. **C.** Anti-NP antibody titers were determined by ELISA 28 days post-vaccination. **E,F.** Anti-NP antibody titer determined 2 weeks postboost by ELISA in all cohorts, and the fold change from the day 28 titer determined. **E.** Fold change in anti-NP titer for animals primed with 10mg of bead-conjugated NP-Ova. **F.** Fold change in anti-NP titer for animals primed with 100mg of bead-conjugated NP-Ova. Each symbol represents an individual animal. Composite of 2 experimental cohorts. Studies conducted in TH-iDTR, neurologically-intact controls included cre- iDTR littermates treated with DT and TH-iDTR mice treated with PBS. *p<0.05, **p<0.01.

If nerve-dependent remodeling is required to concentrate large antigens in the B cell follicles, then increasing the total amount of antigen given in the primary vaccination should rescue the B cell response to large antigens in the absence of nerves. However, there may be differences in other biochemical properties between Ova and KLH that lead to disparity in how these antigens are deposited in the LN. We coupled NP-Ova with 2μM polystyrene FluoSpheres to generate Big NP-Ova (BNO), and vaccinated mice in the footpad with either 10μg or 100μg of protein in the DT-treated footpad (Fig. 4A). Two weeks after vaccination, we found that germinal center formation was impaired in nerve-depleted mice if they were vaccinated with our standard protein antigen concentration of 10μg (Fig. 4B). However, if we vaccinated mice with 100μg of protein, then germinal center formation was restored in nerve-depleted animals (Fig. 4B).

Increasing the amount of antigen in the initial priming also rescued the serological response in nervedepleted mice. In a separate cohort, mice were boosted in the contralateral footpad 28 days after priming regular NP-Ova without bead conjugation, and the anti-NP antibody response evaluated just before before and 2 weeks post-boost (Fig. 4A). Nerve-depleted mice vaccinated with 10μg of BNO during the primary response had a similar serological response to nerve-depleted mice vaccinated with 10μg of NP-KLH; the primary anti-NP response was equivalent (Fig. 4C), while the nerve-depleted cohorts had poor secondary antibody responses compared to nerve-intact mice (Fig. 4D). As expected, mice vaccinated with 100μg of BNO in the primary response had higher titers of anti-NP antibodies 28 days post-vaccination compared to mice vaccinated with 10μg of BNO, irrespective of nerve status (Fig. 4C). However, when nerve-depleted cohorts were subsequently challenged, the recall antibody response in the nerve-depleted cohorts was equivalent to nerve-intact mice (Fig. 4E), indicating increasing the amount of antigen 10 fold is sufficient to fully rescue the B cell response. Collectively, these results demonstrate that adrenergic nerves mediate the accumulation of large antigens in developing germinal centers to promote B cell mediated immunity.

We hypothesize that the remodeled trabeculae allow large macromolecules access to the conduit system, however neuronal signaling may instead alter the permeability of the conduit filters, as both FRCs and LECs express adrenergic receptors^27^. Future studies to determine what signals drive this remodeling and how long these structures remain in this “open” conformation will provide insight that can be used for vaccine design. Lymph nodes also serve as traps for pathogens to limit their dissemination throughout the body^28^. Failure to efficiently trap and control a pathogen in the lymph node leads to lymphatic and bloodstream dissemination^29^. Whether this nerve-mediated remodeling of trabeculae plays a role in trapping of pathogens has yet to be determined. These findings are particularly important for patients with peripheral nerve disorders, including neuropathy, fibromyalgia, and paralysis, as patients with these disorders are susceptible to recurrent severe infections^6, 7, 8^. Our studies would indicate this is due, in part, to a failure to prime adequate memory responses.

## Materials and Methods

Experimental Design. The objective of this study was to understand why adrenergic nerve depletion impairs germinal center responses. We used a local depletion mouse model to specifically ablate adrenergic nerves in the draining popliteal lymph node, eliminating potential effects of systemic adrenergic depletion. We used immunofluorescence staining and flow cytometry to quantify germinal center formation in the presence or absence of nerves. We used fixable flourescent dextran deposition to evaluate the size of different molecules that are capable of entering the lymph node paired with a modified gomori’s reticulum stain to visualize the collagen fiber conduit system and trabeculae in serial sections. Deposition of flourescent antigens in the lymph node was determined in freshly frozen tissue sections. Data were pooled from multiple experiments unless otherwise specified. All data points and n values reflect biological replicates.

Mice and nerve depletions. C57Bl6/J (RRID:IMSR_JAX:000664), TH-cre (RRID:IMSR_JAX:008601, B6.Cg-7630403G23Rik^Tg(Th−cre)1Tmd^/J), and iDTR (RRID:IMSR_JAX:007900, C57BL/6-Gt(ROSA)26Sor^tm1(HBEGF)Awai^/J) mice were purchased from Jackson Laboratories. TH-cre mice were crossed with iDTR animals to generate TH-iDTR mice as well as cre- iDTR littermate controls. All mice were treated for nerve depletion at 6–8 weeks of age. For local depletion of adrenergic nerves, mice were injected with 0.1 ng/g of diphtheria toxin (DT) in the footpad 14 and 16 days prior to vaccination. This treatment dose was sufficient to drive depletion of TH+ nerve fibers in the draining popliteal lymph node, but not in the draining inguinal lymph node or the non-draining popliteal lymph node (Fig.S1). Control mice included cre- iDTR mice treated with DT, and TH-iDTR mice injected with PBS in the footpad. No difference was observed in any assays described for cre- iDTR treated with DT or TH-iDTR mice treated with PBS. For complete sympathectomy experiments, sympathectomy was carried out by 6hydroxydopamine (6OHDA) treatment as previously described^30, 31^. Control mice were treated with vehicle alone. Mice were infected or vaccinated 2 weeks after the final 6-OHDA or vehicle treatment. Experiments were conducted in both male and female cohorts, and no sex-dependent differences in response were observed. All animal experiments adhered to University of Oklahoma Health Center IACUC guidelines.

Vaccinations and infections. Mice were challenged with either vaccination or S. aureus infection 2 weeks after the final nerve depletion treatment or mock-treatment. For vaccination experiments, 10ug of antigen 4-Hydroxy-3-nitrophenylacetyl-Keyhole Limpet Hemocyanin (NP-KLH) (Biosearch cat#N5060–5) or 10ug of antigen NP-Ovalbumin (NP-OVA) (Biosearch cat#N5051–10) was diluted in PBS was mixed 1:1 with Alhydrogel (InVivoGen cat# vac-alu-250) for 25μL of vaccine per mouse. Vaccine was then mixed with a small magnetic stir bar for 30 minutes to ensure a homogenous vaccine. Mice were then anesthetized with isoflurane and 25μL of vaccine injected into the footpad. For fluorescently labeled antigen experiments, 10μg of FITC-conjugated Ovalbumin (Invitrogen cat# O23020) was mixed with 2μg of NP-KLH conjugated to 0.02μM Crimson carboxylate-modified microspheres (Invitrogen cat# F8782). NP-KLH was covalently linked to the 0.02μM Crimson microspheres as per the manufacturer’s instructions using 1-Ethyl-3(3-dimethylaminopropyl)carbodiimide (EDAC), and the protein concentration determined after conjugation by Lowry assay using the DC Protein Assay Kit (Bio Rad, Hercules CA). For BIG-NP-Ova, NP-Ova was covalently linked to 2μM Crimson carboxylate-modified microspheres (Invitrogen cat# F8825) as per the manufacturer’s instructions, and protein concentration determined by Lowry assay. Protein standards were prepared by diluting bovine serum albumin in PBS. Standards and sample were quantified as per the manufacturer’s instructions and the optical density determined using a SpectraMax ABS plate reader (Molecular Devices, San Jose, CA). Protein concentration of red NP-KLH was determined using the standard curve of the protein standards. The FITC-Ova and Red-KLH in PBS were diluted in PBS and mixed 1:1 with alhydrogel (InvivoGen, San Diego CA) and injected in 25mL volume in the footpad.

For infection experiments, *S. aureus* strain UAMS-1 was streaked onto tryptic soy agar (TSA) and grown overnight. Single colonies were selected and then grown overnight in tryptic soy broth (TSB). On the day of infection, 100mL of TSB was inoculated with overnight culture, and bacteria were grown until they entered log phase growth as measured by absorbance 600 readings on a spectrophotometer (BioRad). The colony forming units (CFU) per mL was estimated from the A600 reading. Bacteria were diluted into sterile PBS to obtain inoculum to infect mice with ~ 10^5^ CFU in a volume of 25mL. All animals in an experimental cohort were infected with the same inoculum. The inoculum was plated on TSA and the actual infectious dose determined the following day. The range of infectious inoculum for primary infection across all experiments is 1–5×10^5^ CFU of *S. aureus*.

Flow Cytometry. For germinal center and Tfh staining, draining popliteal lymph nodes were isolated 14 days post-vaccination and dissociated through a 70μM filter to generate a single cell suspension. Single cell suspensions were stained for Fas (RRID:AB_2728202, SA367H8, 1:200), GL-7 (RRID:AB_2800677, GL7, 1:200), IgD (RRID:AB_10643423, 11–26c.2a, 1:150), CD38 (RRID:AB_10613468, 90, 1:150), CD19 (RRID:AB_11218994, 6D5, 1:200), B220 (RRID:AB_2572109, RA3–6B2, 1:200) for germinal centers or with CD44 (RRID:AB_312959, IM7, 1:150), CD62L (RRID:AB_313099, MEL-14, 1:150), CXCR5 (RRID:AB_2562208, L138D7, 1:200), PD-1 (RRID:AB_2566548, RMP1–30, 1:200), ICOS (RRID:AB_2535292, 7E.17G8, 1:150, eBioscience), and CD4 (RRID:AB_312713, RM4.5, 1:200) for Tfh on the cell surface in PBS. After surface staining, cells were fixed and permeabilized for 1 hour at 4°C using the eBioscience transcription factor fixation kit as directed (Invitrogen 00-5521-00). Cells were stained intracellularly in perm wash for Ki67 (RRID:AB_11151330, clone SolA15, 1:200, eBioscience) and Bcl-6 (RRID:AB_2562152, 7D1, 1:150) for germinal center B cells, or stained intracellularly with Bcl-6 alone for Tfh stains. Antibodies purchased from Biolegend unless otherwise specified. For tyrosine hydroxylase staining following DT depletions, cells were stained for CD45 (RRID:AB_2650656, 30-F11, 1:200), CD4 (RRID:AB_312715, 1:200), CD8 (RRID:AB_312747, 53 − 6.7, 1:200), CD19 (RRID:AB_313643, 1:200) F4/80 (RRID:AB_893478, BM8, 1:200) and CD11b (RRID:AB_2572122, M1/70, 1:200) and stained for live cells using Zombie Aqua (Biolegend 423101). After surface staining cells were fixed with the eBioscience transcription factor fixation kit, and stained for TH (RRID:AB_325653, 1:200) for 40 minutes. Cells were washed in permabilization buffer and stained with a secondary AF488 labeled Donkey anti-Rabbit IgG to detect the anti-TH antibody (RRID:AB_893531, 1:100). Macrophages were identified as CD45+, CD4/CD8/CD19-, CD11b+, F4/80+. All samples were acquired on a Cytek Aurora full spectrum cytometer (Cytek Biosciences, Bethesda MD.), and samples analyzed using FlowJo software (FlowJo, Ashland, OR.).

ELISA. Immuno plates (Nunc, Thermo Scientific, catalog number 442404) were coated with 10 μg/mL with NP-bovine serum albumin (NP-BSA). Plates were coated with either NP(2)-BSA (BioSearch Technologies cat#N-5050XL-10) or NP(28)-BSA (BioSearch Technologies cat#N-5050H-10) overnight at 4°C, subsequently plates were blocked with 1% BSA in PBS-T for 2 hours at room temperature (RT). Mouse sera was 2-fold serially diluted in PBS-T and loaded into coated enzyme-linked immunosorbent assay (ELISA) plates. Samples were incubated in the coated ELISA plates overnight at 4°C. Wells were washed with PBS-T and incubated for 1 hour with horseradish peroxidase–conjugated goat antimouse IgG1 (1:10,000) (Southern Biotech cat#1070–05). Wells were washed again with PBS-T and developed for 5 minutes at RT with 2,2'-Azino-bis(3-ethylbenzthiazoline-6-sulfonic acid) (ABTS) substrate (SeraCare Cat#5120–0043). ABTS reactions were stopped with 10% wt/vol sodium dodecyl sulfate in water. Endpoint antibody titers were measured by determining the optical density at 405 nm. Antibody titers are reported as the dilution of sera that is 2 standard deviations above the blank wells.

Enzyme-linked immunosorbent spot (Elispot). Multiscreen ELISpot wells (Millipore, Bedford, MA) were prepared for antigen coating by incubating with 35% v/v ethanol for 30 seconds and washed twice with PBS. The plates were coated with anti-mouse IgG (BioLegend cat#405301), NP(2)-BSA, or NP(28)-BSA (10 μg/mL final concentration) and incubated overnight at 4°C. Plates were washed 3 times with PBS and blocked with RPMI 1640 containing 10% FBS for 2 h at room temperature. Isolated bone marrow cells were plated 1×10^4^ cells per well for total IgG and 1×10^5^ cells per well for other coating antigens. Plates were incubated for 18 hours at 37°C and 5% CO_2_. Plates were washed 5 times with PBS and blotted to remove excess PBS. Biotinylated anti-mouse IgG (1:1000) (BioLegend cat#405303) diluted in PBS was added to wells and incubated at room temperature (RT) for 2 hours. Plates were then washed 5 times with PBS as done previously. Streptavidin-ALP was added to wells (1:1000) (MABTECH) and incubated at RT for 1 hour. Plates were washed as described and developed with 5-bromo-4-chloro-3-indolyl phosphate and nitro blue tetrazolium (NCIP/NBT-plus) (MABTECH). Plates were incubated with NCIP/NBT-plus, light protected, for 20 minutes. Plastic backing was removed from plate and plate was washed vigorously in DI water for 10 minutes to remove any NCIP/NBT-plus.

Dextran administration, trabeculae staining, and quantification. 5 minutes prior to euthanasia mice were injected with 100mg of fluorescent dextran in the footpad. In experiments with both high and low molecular weight dextrans, both dextrans were mixed 1:1 and 100mg of each high- and low-molecular weight dextrans were injected. After euthanasia lymph nodes were isolated and immediately fixed in 10% neutral buffered formalin. In some experiments mice were perfused with formalin prior to lymph node isolation and fixation. Lymph nodes were embedded in paraffin wax, and 4μM serial sections obtained. Discontinuous sections a minimum of 16μM apart were selected for trabeculae staining utilizing a modified gomori’s staining kit (ScyTek Laboratories). Slides were rehydrated and stained as per the manufacturer’s instructions. After staining slides were dehydrated, cleared in xylene, and coverslipped using Canada Balsam (ThermoFisher). Slides were scanned at 20X magnification using either an Axioscan 7 (Zeiss) or VS200 (Olympus) slide scanner. Reticulin fiber structures in the follicular areas of the lymph node were measured using either Zen 3.9 software for sections scanned on the Axioscan or OlyVIA software for sections scanned on the VS200. Preference was given to reticulin fiber structures that extended to the subcapsular sinus. Structures that appeared to surround blood vessels or lymphatic vessels due appearing as open circles were excluded from the measurements. Between 30–90 individual fiber structures were measured in each section. The average of all trabeculae diameters for each section of lymph node was determined and visualized using Prism software (Graphpad).

Immunoflourescence staining and quantification of FITC-Ova and Red-KLH in tissue sections. Lymph nodes were isolated 7 days post-vaccination, embedded in OCT, and sectioned at 6–10μM sections. A minimum of 6 discontinuous sections were stained for DAPI and imaged using a VS200 slide scanner (Olympus) at 10X. Areas in the B cell follicle with low DAPI staining were identified as putative germinal centers and scanned at 40X resolution. Within the same experimental cohort identical exposure times were used to image DAPI, FITC, and Cy5 at both 10X and 40X. Images were evaluated for the same germinal center in multiple sections. Consecutive sections were permeabilized with 0.3% Triton X-100 in PBS for 15 minutes at room temperature. Sections were washed and then blocked with 1% bovine serum albumin and 10% normal goat serum (Southern Biotech) in PBS for 30 minutes. Sections were stained with biotinylated anti-IgD (RRID:AB_466860, 11–26c.2a, Invitrogen, 1:100) and anti-GL7 (RRID:AB_2800675, GL7, Biolegend, 1:50) in blocking buffer overnight at room temperature.. Sections were washed in PBS then stained with Streptavidin-AF750 (Invitrogen #S21384, 1:800) in blocking buffer for 1 hour at room temperature, washed and stained with DAPI, and coverslipped with Prolong Gold (Invitrogen #P36930). In ImageJ, the fluorescence intensity of FITC and Cy5 was determined in the germinal center area. For multiple images of the same germinal center, the average fluorescence intensity for both FITC and Cy5 was determined. For FITC-KLH immunized cohorts, the sections were stained for IgD and GL-7 as described above, and the fluorescence intensity of FITC determined in the germinal centers.

Statistical analyses. Statistical analyses were performed using GraphPad Prism. For comparisons between two normally distributed groups, a student’s T test was performed. For non-normally distributed groups, a Mann-Whitney U test was performed between two groups.

## Supplementary Material

Supplementary Files

This is a list of supplementary files associated with this preprint. Click to download.

- ExtendedDataFigures.docx

## Data Availability

All data are available in the main text or the Extended Data materials.
